# „Intensive Care Unit-Acquired Weakness“: Eine bundesweite Umfrage zu Diagnostik, Monitoring und Therapiestrategien auf deutschen Intensivstationen

**DOI:** 10.1007/s00101-022-01089-9

**Published:** 2022-02-02

**Authors:** Felix Klawitter, Stefan J. Schaller, Martin Söhle, Daniel A. Reuter, Johannes Ehler

**Affiliations:** 1 Klinik und Poliklinik für Anästhesiologie und Intensivtherapie, Universitätsmedizin Rostock, Rostock, Deutschland; 2 Klinik für Anästhesiologie m. S. operative Intensivmedizin (CVK/CCM), Charité – Universitätsmedizin Berlin, Corporate member of Freie Universität Berlin and Humboldt-Universität zu Berlin, Berlin, Deutschland; 3 Klinik für Anästhesiologie und Operative Intensivmedizin, Universitätsklinikum Bonn, Bonn, Deutschland

**Keywords:** Intensivmedizin, „Critical-illness“-Polyneuropathie, „Critical-illness“-Myopathie, Kritische Erkrankung, Umfragen und Fragebogen, Crit care medicine, Critical illness, Critical illness polyneuropathy, Critical illness myopathy, Surveys and questionnaires

## Abstract

**Hintergrund:**

Die „Intensive Care Unit-Acquired Weakness“ (ICU-AW) ist eine der häufigsten Ursachen für eine neuromuskuläre Dysfunktion in der Intensivmedizin. Gegenwärtig fehlen evidenzbasierte Empfehlungen zur Diagnostik, zum Monitoring und zu therapeutischen Maßnahmen.

**Ziel der Arbeit:**

Die Erfassung des derzeitigen Vorgehens bei Diagnostik, Monitoring und präventiven und therapeutischen Ansätzen bei der ICU-AW auf deutschen Intensivstationen.

**Material und Methoden:**

Onlinebefragung von 448 Mitgliedern des Wissenschaftlichen Arbeitskreises Intensivmedizin (WAKI) und des Wissenschaftlichen Arbeitskreises Neuroanästhesie (WAKNA).

**Ergebnisse:**

Insgesamt wurden 68/448 (15,2 %) Fragebogen ausgewertet. Bei 13,4 % (9/67) der Befragten existiert ein strukturiertes diagnostisches Vorgehen zur Detektion der ICU-AW. Für Screening (60/68; 88,2 %) und Verlaufsbeurteilung (57/65; 87,7 %) wird die klinische Untersuchung präferiert. Etablierte Scores, wie der „Medical Research Council sum score“ (MRC-SS) spielen für Screening und Verlaufskontrolle der ICU-AW eine untergeordnete Rolle (7/68; 10,3 % und 7/65; 10,8 %). Mobilisation (45/68; 66,2 %) und Sedativareduktion (38/68; 55,9 %) stellen die häufigsten präventiven und therapeutischen Ansätze dar. Ein Mangel an Physiotherapeuten (64/68; 94,1 %) und Pflegekräften (57/68; 83,8 %) wird als Hauptdefizit bei der Versorgung von Patienten mit ICU-AW identifiziert. Insgesamt 91,2 % (62/68) der Befragten befürworten die Erstellung evidenzbasierter Empfehlungen zur Diagnostik, zum Monitoring und zu therapeutischen Ansätzen bei ICU-AW.

**Diskussion:**

Ein einheitliches Konzept für Diagnostik, Monitoring, Prävention und Therapie der ICU-AW auf deutschen Intensivstationen fehlt weitgehend. Innovative diagnostische Ansätze könnten in Zukunft helfen, Patienten mit einem hohem Risiko für eine ICU-AW frühzeitig zu detektieren, präventive Maßnahmen einzuleiten sowie wertvolle prognostische Informationen zu gewinnen.

**Zusatzmaterial online:**

Die Online-Version dieses Beitrags (10.1007/s00101-022-01089-9) enthält den der Studie zugrunde liegenden Fragebogen.

## Abstract (en)

**Background:**

Intensive care unit-acquired weakness (ICU-AW) is one of the most frequent causes of neuromuscular dysfunction in intensive care medicine. To date no evidence-based recommendations exist for the diagnostics, monitoring or further intensive care treatment.

**Objective:**

To evaluate the current clinical practice of diagnostics, monitoring and treatment strategies of ICU-AW on intensive care units in Germany.

**Material and methods:**

We conducted an online survey with a self-designed questionnaire and invited 448 members of the Scientific Working Group for Intensive Care Medicine (WAKI) and the Scientific Working Group for Neuroanesthesia (WAKNA) to participate.

**Results:**

A total of 68/448 (15.2%) questionnaires were analyzed. Of the participants 13.4% (9/67) stated that a structured diagnostic approach for the detection of ICU-AW is applied in their units. The clinical examination was the preferred method for screening (60/68; 88.2%) and follow-up (57/65; 87.7%). Scores, such as the Medical Research Council sum score (MRC-SS) seem to be less important for the screening (7/68; 10.3%) and follow-up assessment (7/65; 10.8%). Mobilization with physiotherapy (45/68; 66.2%) is the most common strategy applied to treat ICU-AW. A lack of physiotherapists (64/68; 94.1%) and intensive care nurses (57/68; 83.8%) are the main deficits identified in the care of patients with ICU-AW. The majority of the study participants (62/68; 91.2%) would welcome evidence-based guidelines for diagnostics, monitoring and treatment approaches in ICU-AW.

**Discussion:**

To date comprehensive recommendations for diagnostics, monitoring, prevention and treatment of ICU-AW are still lacking in German intensive care units. The introduction of new diagnostic approaches could help to detect ICU-AW and therefore to initiate earlier preventive and treatment approaches.

## Hinführung

Die Intensive Care Unit-Acquired Weakness (ICU-AW) gilt als eine der häufigsten Ursachen für eine neuromuskuläre Dysfunktion beim Intensivpatienten und ist sowohl mit einer erhöhten Morbidität und Letalität als auch mit einem schlechteren Langzeit-Outcome assoziiert. Bisher existieren keine einheitlichen Empfehlungen zur Diagnostik, zum Monitoring oder zu präventiven und therapeutischen Strategien während der Intensivbehandlung. Wie wird eine der häufigsten intensivmedizinischen Komplikationen also im täglichen klinischen Alltag aktuell auf deutschen Intensivstationen diagnostiziert und behandelt?

## Hintergrund

Die ICU-AW ist definiert als eine neu erworbene neuromuskuläre Schwäche, die sekundär im Rahmen einer kritischen Erkrankung auftritt und durchschnittlich bis zu 40 % der Intensivpatienten betreffen kann [3]. Hierbei ist die ICU-AW mit einer verlängerten Beatmungsdauer [11], einer längeren Intensivbehandlung [1] sowie einer erhöhten Morbidität und Letalität verbunden [24]. Pathophysiologisch kann der ICU-AW eine Myopathie („Critical-illness“-Myopathie, CIM), eine Neuropathie („Critical-illness“-Polyneuropathie, CIP) oder ein gemischtes Krankheitsbild („Critical-illness“-Polyneuromyopathie, CIPNM) zugrunde liegen. Klinisch zeigt sich häufig eine schlaffe Tetraparese mit reduzierten oder erloschenen Muskeleigenreflexen unter Aussparung der kraniofazialen Muskulatur [12]. Ist zusätzlich die Atemmuskulatur betroffen (v. a. das Zwerchfell), verschlechtert sich das Langzeit-Outcome der Patienten zusätzlich [20]. Die Diagnose einer ICU-AW wird dabei im klinischen Kontext der Intensivbehandlung durch das Vorliegen der typischen Symptome, kombiniert mit einem „Medical Research Council sum score“ (MRC-SS) < 48, und nach Ausschluss anderer Ursachen gestellt [25]. Aktuelle Evidenz weist darauf hin, dass bereits ein MRC-SS < 55 mit einem schlechterem Behandlungsergebnis assoziiert ist [27]. Darüber hinaus wurden unterschiedliche diagnostische, therapeutische und präventive Ansätze zu Detektion, Behandlung und Prognoseabschätzung der ICU-AW publiziert [6, 10, 12, 13, 16, 21, 28].

Dennoch fehlen bisher einheitliche, evidenzbasierte Empfehlungen für die Diagnostik, das Monitoring sowie für präventive und therapeutische Maßnahmen, sodass die Autoren eine große Heterogenität in der Art und dem Umfang der diagnostischen und therapeutischen Ansätze auf deutschen Intensivstationen vermuten. Zur Evaluation der aktuellen klinischen Praxis bei der ICU-AW wurde daher eine nationale Umfrage zu diesem Thema durchgeführt.

## Studiendesign und Untersuchungsmethoden

Auf der Grundlage der aktuellen wissenschaftlichen Literatur wurde für die vorliegende Studie ein Fragebogen mit 36 Items entwickelt. Die Studie wurde als Onlineumfrage gemäß der gültigen Datenschutzrichtlinie und nach positivem Ethikvotum der Ethikkommission der Universität Rostock (Registriernummer A 2021-0030) durchgeführt. Zur Erstellung des Onlinefragebogens wurde die Evaluationssoftware EvaSys® (Version 8.0, Fa. Electric Paper Evaluationssysteme GmbH, Lüneburg, Deutschland) genutzt. Über den Mail-Verteiler der Deutschen Gesellschaft für Anästhesiologie und Intensivmedizin (DGAI) wurden die Mitglieder des Wissenschaftlichen Arbeitskreises Neuroanästhesie (WAKNA) und des Wissenschaftlichen Arbeitskreises Intensivmedizin (WAKI) via E‑Mail zur anonymen Umfrageteilnahme eingeladen. Doppelte Versendungen an Mitglieder beider Arbeitskreise wurden ausgeschlossen. Die Rückverfolgung einzelner Studienteilnehmer war hierbei technisch ausgeschlossen worden. Es wurde einmalig via E‑Mail an die Studienteilnahme erinnert. Die statistische Auswertung erfolgte mit IBM SPSS Statistics (Version 25, Fa. IBM Corp., Armonk, NY, USA). Kategoriale Variablen wurden mit dem Chi-Quadrat-Test bzw. dem Exakten Test nach Fisher verglichen. Das Signifikanzniveau für einen statistischen Test wurde auf einen *p*-Wert < 0,05 festgelegt.

## Ergebnisse

### Demografische Basisdaten

Im Zeitraum von März bis April 2021 wurden insgesamt 448 Mitglieder des WAKI und des WAKNA zur Umfrageteilnahme eingeladen, wobei 68 (15,2 %) Mitglieder den Fragebogen beantworteten. Die allgemeinen demografischen Basisdaten der Studienteilnehmer und deren Klinik zeigt Tab. 1. Die Mehrzahl der Befragten war als Fach- oder Oberarzt mit zumeist über 10 Jahren Berufserfahrung in einem universitären Maximalversorger mit einer Kapazität bis zu 50 Intensivbetten auf einer perioperativen Intensivstation (ITS) tätig.

**Table 1:** 

**Verteilung nach Versorgungsstufen**	**Absolut (n/68)**	**Relativ (%)**
Krankenhaus der Grundversorgung	6	8,8
Krankenhaus der Schwerpunktversorgung	11	16,2
Krankenhaus der Maximalversorgung	10	14,7
Krankenhaus der Maximalversorgung (Universitätsklinik)	41	60,3
**Verteilung nach Anzahl der Intensivbetten**	**Absolut (n/67)**	**Relativ (%)**
< 10	2	3
10–20	18	26,9
21–50	41	61,2
> 50	6	9
**Verteilung nach Art der Intensivstation (ITS)**	**Absolut (n/68)**	**Relativ (%)**
Perioperative ITS	43	63,2
Internistische ITS	0	0
Neurologische ITS	0	0
Interdisziplinäre pädiatrische ITS	0	0
Interdisziplinäre ITS	25	36,8
**Verteilung nach Klinikposition**	**Absolut (n/67)**	**Relativ (%)**
Klinikdirektor/Chefarzt	13	19,4
Oberarzt	32	47,8
Facharzt	16	23,9
Weiterbildungsassistent	6	9
**Verteilung nach Jahren Berufserfahrung**	**Absolut (n/68)**	**Relativ (%)**
< 5	12	17,6
5–10	13	19,1
> 10	43	63,2

Darüber hinaus beschäftigten sich 20,6 % (14/68) der Studienteilnehmer an ihrer Klinik wissenschaftlich mit dem Thema der neuromuskulären Schwäche des Intensivpatienten. In 17,6 % (12/68) der Fälle wurde im vergangenen Jahr eine klinikinterne Fortbildung zu dem Thema neuromuskuläre Störung und assoziierte Komplikationen durchgeführt.

### Diagnostik zur Erfassung der ICU-AW

**Figure 1:**
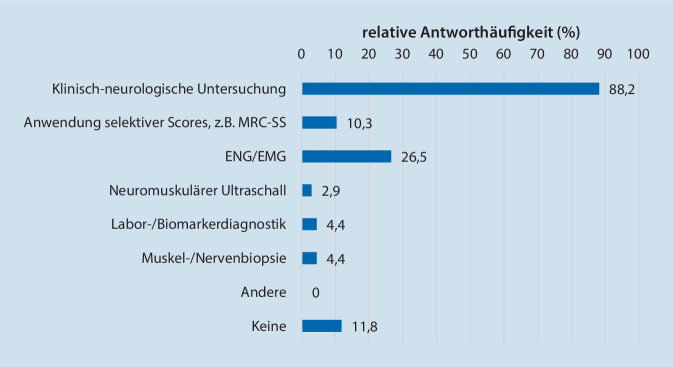

Die Ergebnisse des Abschnitts „Diagnostik der ICU-AW“ zeigt Tab. 2. Insgesamt liegt in 13,4 % (9/67) ein strukturiertes diagnostisches Konzept zur Detektion einer ICU-AW (z. B. in Form einer hausinternen „standard operating procedure“ - SOP) vor. Dabei wird eine auf der Intensivstation neu erworbene neuromuskuläre Schwäche am häufigsten als CIP (51/68; 75 %) oder CIM (46/68; 67,6 %) bezeichnet. Die klinisch-neurologische Untersuchung stellt mit 88,2 % (60/68) die am häufigsten eingesetzte Methode für ein systematisches Screening zur Detektion der ICU-AW dar (Abb. 1). Weniger häufig eingesetzt wurden die Elektroneurographie - ENG/ die Elektromyographie - EMG (18/68), Scores (z. B. MRC-SS 7/68), Labor- und Biomarkerdiagnostik (3/68), Muskel- und Nervenbiopsien (3/68) oder ein neuromuskulärer Ultraschall (2/68). Keine Diagnostik erfolgt bei 8/68 Befragten. Eine Übersicht zur Verteilung von Methoden zu Diagnostik und Screening gibt Tab. 2. Am häufigsten wird auf das Vorliegen einer ICU-AW gescreent, wenn der Patient über einen längeren Zeitraum eine reduzierte oder keine motorische Eigenaktivität zeigt (79,4 %, 54/68). Ein routinemäßiges Screening wird laut den Befragten in 35,3 % (24/68) der Fälle durchgeführt und in 26,5 % (18/68) der Fälle zumindest einmal täglich. Sofern ein Screening durchgeführt wird, wird dies aktuell am häufigsten vom ärztlichen Personal übernommen (85,5 %; 47/55).

**Table 2:** 

**Verwendete Begriffe für eine neu erworbene neuromuskuläre Dysfunktion (Mehrfachantworten möglich)**	**Absolut (n/68)**	**Relativ (%)**
CIP	46	67,6
CIM	51	75,0
CIPNM	13	19,1
ICU-AW	36	52,9
Andere	0	0
Keine	0	0
**Klinische Szenarien, bei denen auf das Vorliegen einer ICU-AW gescreent wird (Mehrfachantworten möglich)**	**Absolut (n/68)**	**Relativ (%)**
Routinemäßig, im Rahmen der täglichen klinischen Untersuchung	24	35,3
Wenn Grunderkrankung und Erkrankungsschwere eine ICU-AW wahrscheinlich machen	44	64,7
Patient zeigt über längeren Zeitraum eine reduzierte oder keine motorische Eigenaktivität	54	79,4
Nach einem erfolglosen Weaning	39	57,4
Andere	1	1,5
Keine	23	4,4
**Zeitliche Abstände, in denen auf ICU-AW gescreent wird**	**Absolut (n/68)**	**Relativ (%)**
Einmal pro Intensivaufenthalt des Patienten	9	13,2
Einmal täglich	18	26,5
Einmal pro Schicht	4	5,9
Ein Screening wird nicht durchgeführt	37	54,4
**Wer sollte ein Screening auf ICU-AW durchführen?**	**Absolut (n/66)**	**Relativ (%)**
Pflegepersonal	12	18,2
Ärztliches Personal	35	53
Physiotherapeuten	19	28,8
**Wer führt aktuell das Screening auf ICU-AW durch?**	**Absolut (n/55)**	**Relativ (%)**
Pflegepersonal	2	3,6
Ärztliches Personal	47	85,5
Physiotherapeuten	6	10,9

*ICU-AW* „Intensive Care Unit-Acquired Weakness“, *CIP* „Critical-illness“-Polyneuropathie, *CIM* „Critical-illness“-Myopathie, *CIPNM* „Critical-illness“-Polyneuromyopathie

### Monitoring zur Verlaufskontrolle der ICU-AW

**Figure 2:**
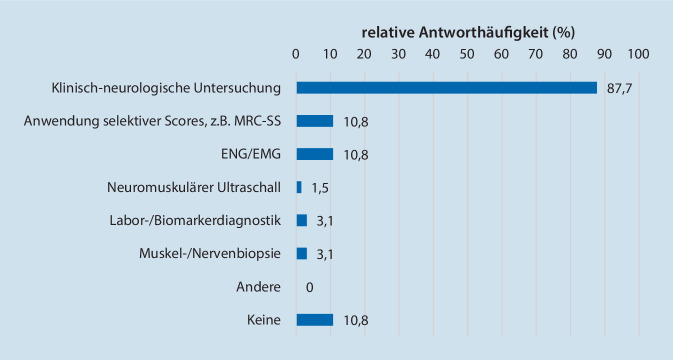

Nach der Diagnosestellung der ICU-AW erfolgt das Hinzuziehen eines neurologischen Konsiliararztes in 54,4 % (37/68) der Fälle, und in 27,9 % (19/68) der Fälle wird eine weiterführende elektrophysiologische Diagnostik durchgeführt (Tab. 3). Für die klinische Verlaufsbeurteilung von Patienten mit ICU-AW wird die klinische Untersuchung von den Studienteilnehmern favorisiert (Abb. 2, 87,7 %; 57/65). Elektrophysiologische Untersuchungen (ENG/EMG) und selektive Scores (z. B. der MRC-SS) werden weniger häufig eingesetzt (jeweils 10,8 %, 7/65). Das Ausmaß der körperlichen Einschränkung von Intensivpatienten wird in 95,6 % (61/65) der Fälle nicht anhand von Scoring-Systemen erfasst (Abb. 2).

**Table 3:** 

**Angewendete weiterführende Diagnostik nach Diagnosestellung einer ICU-AW**	**Absolut (n/68)**	**Relativ (%)**
Elektrophysiologische Untersuchungen (ENG/EMG)	19	27,9
Neuromuskulärer Ultraschall	2	2,9
Muskel‑/Nervenbiopsie	3	4,4
Anmeldung eines neurologischen Konsils	37	54,4
Labor‑/Biomarkerdiagnostik	2	2,9
Andere, hier nicht genannte	0	0
Keine	26	38,2
**Angewendete Scores zur Einschätzung der körperlichen Einschränkung der Intensivpatienten**	**Absolut (n/65)**	**Relativ (%)**
Functional Independence Measure (FIM)	0	0
Physical Function in the ICU Test (PFIT)	1	1,5
Functional Status Score for the ICU (FSS-ICU)	1	1,5
Acute Care Index of Function (ACIF)	0	0
Andere, hier nicht genannte	2	3,1
Keine	61	93,8

*ICU-AW* „Intensive Care Unit-Acquired Weakness“, *EMG* Elektromyographie, *ENG* Elektroneurographie

### Klinisch-therapeutische Konsequenzen

**Figure 3:**
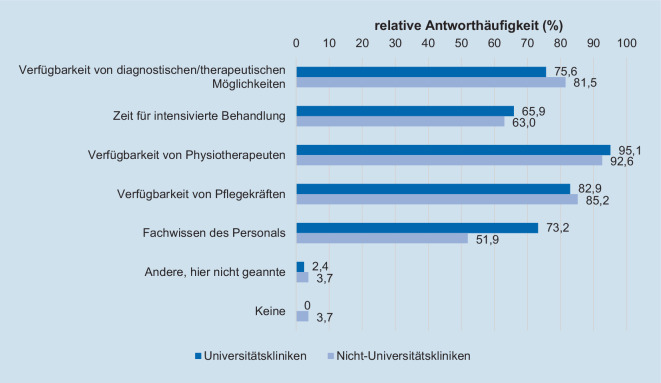

Insgesamt 14,7 % (10/68) der befragten Kollegen gaben an, ein strukturiertes Konzept nach Diagnosestellung einer ICU-AW auf ihrer Intensivstation vorliegen zu haben. Dass zu den häufigsten weiterführenden intensivmedizinischen Konzepten nach Diagnosestellung einer ICU-AW die Einleitung bzw. Intensivierung einer Physiotherapie (66,2 %; 45/68) sowie die Reduktion der Sedativa (55,9 %; 38/68) gehören, zeigt Tab. 4. Dabei kann eine physiotherapeutische Behandlung in 79,4 % (54/68) der Fälle einmal täglich durchgeführt werden. Der Personalmangel an Physiotherapeuten (94,1 %; 64/68) und Pflegekräften (83,8 %; 57/68) sowie das Fehlen von diagnostischen und/oder therapeutischen Möglichkeiten werden als häufigste Defizite in der Versorgung von Intensivpatienten mit ICU-AW aufgeführt (Abb. 3). Gemäß 38,2 % (26/68) der Befragten werden Angehörige von Patienten nicht routinemäßig über mögliche längerfristige körperliche Einschränkungen bei Vorliegen einer ICU-AW aufgeklärt. Erfasst wird die Diagnose einer neu erworbenen neuromuskulären Störung bzw. ICU-AW hingegen zu 98,2 % (56/57) in der Epikrise des Patienten. Insgesamt befürworten 91,2 % (62/68) der Studienteilnehmer die Erstellung einer Leitlinie zu Diagnostik, Monitoring und klinisch-therapeutischen Ansätzen bei ICU-AW.

**Table 4:** 

**Weiterführende intensivmedizinische Maßnahmen nach Diagnosestellung einer ICU-AW (Mehrfachantworten möglich)**	**Absolut (n/68)**	**Relativ (%)**
Einleitung bzw. Intensivierung einer physiotherapeutischen Behandlung	45	66,2
Anwendung neuromuskulärer elektrischer Stimulation	1	1,5
Intensivierte Insulintherapie zur Vermeidung von Hyperglykämien	14	20,6
Vermeidung von Muskelrelaxanzien	25	36,8
Vermeidung von Kortikosteroiden	24	35,3
Reduktion von Sedativa	38	55,9
Andere, hier nicht genannte	3	4,4
Keine	9	13,2
**Häufigkeit der Verfügbarkeit einer physiotherapeutischen Behandlung in der klinischen Routine**	**Absolut (n/68)**	**Relativ (%)**
Mehrmals pro Schicht	4	5,9
Einmal pro Schicht	8	11,8
Einmal täglich	54	79,4
In unregelmäßigen Abständen	2	2,9
Gar nicht	0	0

*ICU-AW* „Intensive Care Unit-Acquired Weakness“

## Diskussion

Mit der vorliegenden Umfrage wurde der aktuelle Stand von Diagnostik, Monitoring und klinisch-therapeutischen Ansätzen bei ICU-AW auf deutschen Intensivstationen untersucht. Hierbei zeigte sich, dass die ICU-AW eine klinische Herausforderung für Intensivstationen aller Versorgungsstufen ist und die eingesetzten diagnostischen und therapeutischen Ansätze heterogen verteilt sind. Nur in den wenigsten Fällen liegt ein strukturiertes Konzept zur Erfassung, zur Verlaufskontrolle und zur weiterführenden Behandlung einer neuromuskulären Schwäche auf der Intensivstation vor. Besonders wertvoll für die Einschätzung der in dieser Studie gewonnenen Einsichten ist die hohe Beteiligung (63,2 %) von Kollegen mit einer langjährigen intensivmedizinischen Berufserfahrung von mindestens 10 Jahren (Tab. 1).

### Diagnostik und Monitoring der ICU-AW

#### Screening und Diagnosestellung

Die ICU-AW ist per Definition eine klinische Diagnose ohne die primäre Anwendung bildgebender, elektrophysiologischer oder laborchemischer Verfahren. Stevens et al. entwickelten einen Leitfaden mit genau definierten Kriterien für die ICU-AW [25]. Demnach ist die systematische Beurteilung der Kraftgrade einzelner Muskelgruppen anhand des MRC-SS, des Muskeltonus und der Reflexstatus bzw. die Abhängigkeit von einer mechanischen Beatmung sowie der Ausschluss anderer Ursachen für die Diagnosestellung erforderlich. Zwar ist die klinisch-neurologische Untersuchung die am häufigsten eingesetzte diagnostische Methode in der Umfrage, jedoch werden validierte Scores, wie der MRC-SS, lediglich in 10,3 % der Fälle erhoben. Da viele Intensivpatienten mit ICU-AW häufig länger sediert und beatmet werden [11], könnte dies die Erhebung des MRC-SS zumindest in der Akutphase einschränken. Auch könnten fehlende Kenntnisse über die korrekte Anwendung hier eine Rolle spielen. Kongruent zu dieser Vermutung war das Ergebnis der in dieser Studie gestellten Testfrage (Zusatzmaterial online: Fragebogen, Frage 5.9, s. Box am Beitragsanfang): lediglich 64,5 % (40/62) der Befragten identifizierten die Aussage, beim MRC-SS würde die Fähigkeit zur Mitarbeit des Patienten keine Rolle spielen, korrekt als die gesuchte Falschantwort. Dies zeigt, dass der MRC-SS in der Routinediagnostik eher unzureichend etabliert ist.

Elektrophysiologische Untersuchungen (ENG, EMG) oder Muskel- und Nervenbiopsien können eine CIP oder CIM zwar erfassen, werden aber womöglich aufgrund der notwendigen technischen Voraussetzungen und des invasiven Vorgehens bisher eher weniger in der klinischen Praxis eingesetzt (Abb. 1). Mögliche Alternativen zur nichtinvasiven und Compliance-unabhängigen Detektion der ICU-AW, wie der neuromuskuläre Ultraschall oder die Bestimmung von Blutbiomarkern sind weiterhin Gegenstand wissenschaftlicher Untersuchungen [14, 17], jedoch noch nicht in der Routinediagnostik etabliert.

Das Screening auf eine ICU-AW wird dabei eher unregelmäßig in Anhängigkeit vom klinischen Gesamteindruck des Patienten durchgeführt (Tab. 2), wodurch die Diagnose jedoch erst verspätet gestellt und sekundär präventive sowie therapeutische Maßnahmen verzögert eingeleitet werden könnten.

#### Monitoring und weiterführende Diagnostik

Um die Diagnose hinsichtlich einer CIP, CIM oder CIPNM zu spezifizieren, sind zusätzliche elektrophysiologische Untersuchungen notwendig [25]. Dabei wird im klinischen Alltag am häufigsten die Diagnose der neuromuskulären Schwäche als CIP (75 %) oder CIM (67,6 %) bezeichnet, jedoch ohne Durchführung der dafür notwendigen Testverfahren: Weder zum initialen Screening (26,5 %) noch in der weiterführenden Diagnostik (27,9 %) wird ein ENG oder EMG primär durchgeführt. Eine gewisse klinische Relevanz hätte die Differenzierung einer ICU-AW jedoch für die Prognoseabschätzung, da Intensivpatienten mit einer CIP eine längere oder unvollständige Rekonvaleszenz zeigen können als Patienten mit einer CIM [9].

Standardisierte Scorings können helfen, physische Einschränkungen der Intensivpatienten zu erfassen und das Outcome abzuschätzen [7], werden in der klinischen Praxis aber so gut wie gar nicht verwendet (Tab. 3). Dies könnte am Umfang und an der zusätzlich vorgesehenen kognitiven Funktionstestung der Items liegen, was die Anwendbarkeit bei prolongierter Beatmung und Sedierung einschränken würde.

### Klinisch-therapeutische Konsequenzen

#### Kontrolle von Risikofaktoren, Frühmobilisation und Physiotherapie

Eine kausale Therapie der ICU-AW ist bisher nicht möglich. Als mögliche Präventions- und Therapieansätze werden die Behandlung der Grunderkrankung (z. B. Sepsis) sowie die Reduktion oder Kontrolle von Risikofaktoren (intensivierte Insulintherapie zur Vermeidung von Hyperglykämien, Sedierungsreduktion, Verzicht auf eine vorzeitige parenterale Ernährung) diskutiert [28]. In unserer Befragung wird die Einleitung bzw. Intensivierung einer physiotherapeutischen Behandlung als wichtigstes therapeutisches Konzept nach Diagnosestellung einer ICU-AW angesehen (Tab. 4), was überwiegend auch in der wissenschaftlichen Literatur bestätigt wird. Der frühzeitige Beginn der Mobilisation scheint hierbei entscheidender zu sein als die Anwendung eines standardisierten Vorgehens [15, 21]. Anekwe et al. [2] und Zhang et al. [30] beschreiben in ihren Übersichtsartikeln ebenfalls einen potenziellen Nutzen frühzeitiger Mobilisierungsmaßnahmen im Hinblick auf Prävention und Outcome-Verbesserung, wohingegen andere Arbeitsgruppen die Datenlage als zu inkonsistent einschätzen, um eine abschließende Bewertung abgeben zu können [6, 8]. Gemäß der vorliegenden Umfrage kann eine physiotherapeutische Behandlung von Patienten mit ICU-AW zumindest einmal täglich realisiert werden, allerdings wäre für Intensivpatienten eine häufigere und intensivere Mobilisation unter physiotherapeutischer Anleitung wünschenswert [5]. Intensität und Frequenz physiotherapeutischer Maßnahmen bei Patienten mit ICU-AW sind als Outcome-Faktoren bisher nicht systematisch untersucht worden [4, 30], jedoch gibt es Hinweise, dass eine individualisierte und zielorientierte Mobilisationsstrategie die Dauer der Intensivbehandlung verkürzen sowie das funktionelle Outcome verbessern könnte [21]. Weitere Studien weisen darauf hin, dass zumindest ein höheres Level an Mobilisierung mit einem besseren Outcome assoziiert ist [18, 22]. Ob Patienten mit elektrophysiologisch vorrangig neuropathischer oder myopathischer Komponente von einer differenzierteren Therapie profitieren könnten, ist bisher nicht geklärt.

#### Neuromuskuläre elektrische Stimulation

Die Anwendung der neuromuskulären elektrischen Stimulation (NMES) hat gemäß unserer Umfrage aktuell einen geringen Stellenwert in der Prävention und Behandlung der ICU-AW. Auch wenn die Datenlage hierzu in einigen Punkten noch inkonsistent ist, zeigen aktuelle randomisierte kontrollierte Studien sowie einige Metaanalysen und Reviews der letzten Jahre überwiegend positive Ergebnisse mit Hinweisen auf eine Verkürzung der Beatmungsdauer, des ITS-Aufenthalts sowie eine Reduktion der Muskelatrophie und des Auftretens einer ICU-AW [13, 23, 26, 29]. Es existieren bisher keine einheitlichen Empfehlungen für die Anwendung einer NMES bei Intensivpatienten, was kongruent zu dem Antwortverhalten der Befragten war.

#### Barrieren und Defizite

Die häufigsten Defizite in der Versorgung von Intensivpatienten mit ICU-AW werden gemäß dieser Befragung in der Verfügbarkeit von medizinischem Personal (Physiotherapeuten und Pflegekräften) sowie in diagnostischen und therapeutischen Möglichkeiten gesehen (Abb. 3). Im klinikübergreifenden Vergleich stimmen Universitäts- und Nichtuniversitätskliniken dabei in ihrem Antwortverhalten weitestgehend überein. So konnte gezeigt werden, dass das Patienten-Outcome direkt vom Pflegeschlüssel beeinflusst ist [19]. Sehr wahrscheinlich ist daher auch eine adäquate personelle Besetzung zur bestmöglichen Versorgung von Patienten mit ICU-AW von großer Bedeutung, da diese Patienten erfahrungsgemäß zeitlich und personell mehr Ressourcen im klinischen Alltag erfordern als Patienten ohne ICU-AW.

Die ICU-AW scheint als Diagnose zwar in den meisten Fällen Eingang in die Epikrise des Patienten zu finden, doch werden Angehörige über die möglichen längerfristigen physischen Beeinträchtigungen häufig nicht informiert. Dabei sollte auch die Familie als wichtige soziale Stütze des Patienten frühzeitig auf die Herausforderungen der nachfolgenden Rehabilitationsphase vorbereitet werden.

Limitierend für die Generalisierbarkeit der vorliegenden Studienergebnisse sind die nur mäßige Umfragebeteiligung von 15,2 % und der Selektionsbias von sehr erfahrenen Kollegen aus dieser Population. Dennoch gibt es zu dieser Thematik bisher keine anderen Daten aus dem deutschen Gesundheitswesen, was für die Notwendigkeit weiterer nationaler Untersuchungen spricht. Ebenfalls wurde nur eine vorselektionierte Kohorte von ärztlichen Kollegen befragt. Interessant wäre auch eine Erhebung über Kenntnisse und den praktischen Umgang von anderen medizinischen Fachkollegen, wie Physiotherapeuten in Akut- und Rehabilitationseinrichtungen.

## Zusammenfassung

Einheitliche Versorgungskonzepte hinsichtlich der Diagnostik, dem Monitoring und der Behandlung von Patienten mit ICU-AW fehlen überwiegend auf deutschen Intensivstationen.

Die in dieser Studie dargelegte methodische Heterogenität sollte Anlass sein, die Evidenz der bereits vorhandenen diagnostischen und therapeutischen Möglichkeiten bei ICU-AW, wie auch von der Mehrheit der Umfrageteilnehmer gefordert, künftig innerhalb einer Leitlinienerstellung zu berücksichtigen und zu bewerten.

## Fazit für die Praxis

- Einheitliche diagnostische und therapeutische Konzepte zur ICU-AW sind auf den meisten deutschen Intensivstationen bisher nicht verfügbar.
- Validierte Testverfahren zur adäquaten Diagnosestellung von ICU-AW, CIP und CIM scheinen derzeit unzureichend angewendet zu werden.
- Der Mangel an personellen Ressourcen wird als größtes Defizit in der Versorgung von Patienten mit ICU-AW angesehen.
- Physiotherapie und Mobilisation werden als wichtigste therapeutische Ansätze bei der ICU-AW wahrgenommen.

## Supplementary Information




