# Supplementary material for: Intensive Care Unit-Acquired Weakness: A nationwide survey on diagnostics, monitoring and treatment strategies on German intensive care units
Source: Anaesthesiologie. 2022 Feb 2;71(8):618–25. [Article in German] doi: 10.1007/s00101-022-01089-9 (PMC9352631; doi:10.1007/s00101-022-01089-9)
Supplement: Supplementary file 1 [file 101_2022_1089_MOESM1_ESM.pdf]

Universitätsmedizin Rostock
Klinik und Poliklinik für Anästhesiologie und

AG NeuroIntens
Intensive Care Unit - Acquired Weakness

Bitte so markieren: ☐ ☒ ☐ ☐ ☐
Korrektur: ☐ ■ ☐ ☒ ☐

1. Einwilligung zur Teilnahme

1.1 **Die Teilnahme an dieser Studie ist freiwillig. Ihre Daten werden anonym behandelt, sodass nach der Auswertung kein individueller Rückschluss möglich ist. Die Datenerhebung wird voraussichtlich bis Juli 2021 abgeschlossen sein.**

☐ Ja, ich bin einverstanden und möchte an dieser Umfrage teilnehmen.

2. Demografische Basisdaten

2.1 **In welchem Bundesland befindet sich Ihre Klinik?**

☐ Baden-Württemberg ☐ Bayern ☐ Berlin
☐ Brandenburg ☐ Bremen ☐ Hamburg
☐ Hessen ☐ Mecklenburg-Vorpommern ☐ Niedersachsen
☐ Nordrhein-Westfalen ☐ Rheinland-Pfalz ☐ Saarland
☐ Sachsen ☐ Sachsen-Anhalt ☐ Schleswig-Holstein
☐ Thüringen

2.2 **Bitte ordnen Sie Ihre Klinik in eine der folgenden Versorgungsstufen ein:**

☐ Grundversorgung ☐ Schwerpunktversorgung ☐ Maximalversorgung
☐ Maximalversorgung (Universitätsklinik)

2.3 **Bitte geben Sie die Gesamtzahl der Intensivbetten auf Ihrer Intensivstation an.**

☐ <10 ☐ 10-20 ☐ 21-50
☐ >50

2.4 **Bitte ordnen Sie Ihre Intensivstation gemäß dem primären Behandlungsspektrum in <u>eine</u> der nachfolgenden Kategorien ein.**

☐ Perioperative Intensivstation ☐ Internistische Intensivstation ☐ Neurologische Intensivstation
☐ Interdisziplinäre pädiatrische Intensivstation ☐ Interdisziplinäre Intensivstation

2. Demografische Basisdaten [Fortsetzung]

2.5 **Welche Position vertreten Sie auf Ihrer Intensivstation / in Ihrem Klinikum?**

- ☐ Klinikdirektor / Chefarzt
- ☐ Oberarzt
- ☐ Facharzt
- ☐ Weiterbildungsassistent

2.6 **Wie viele Jahre Berufserfahrung haben Sie insgesamt in der Intensivmedizin?**

- ☐ <5
- ☐ 5-10
- ☐ >10

2.7 **Beschäftigen Sie sich an Ihrer Klinik forschungsmäßig mit dem Thema neuromuskuläre Schwäche des Intensivpatienten bzw. ICU-AW?**

- ☐ Ja
- ☐ Nein

3. Diagnostik der ICU-AW

3.1 **In der wissenschaftlichen Literatur sind eine Vielzahl von Begriffen zur Beschreibung neu erworbener neuromuskulärer Störungen bei Intensivpatienten bekannt.**

Welche der nachfolgenden Begriffe zur Beschreibung neu erworbener neuromuskulärer Störungen verwenden Sie auf Ihrer Intensivstation?

Bitte wählen Sie alle zutreffenden Antwortmöglichkeiten aus.

- ☐ Critical Illness Myopathie (CIM)
- ☐ Critical Illness Polyneuropathie (CIP)
- ☐ Critical Illness Polyneuromyopathie (CIPNM)
- ☐ Intensive Care Unit - Acquired Weakness (ICU-AW)
- ☐ Andere, hier nicht genannte
- ☐ Keine

3.2 **Wenn Sie eine andere Begrifflichkeit für die auf Ihrer Intensivstation erfassten, neu erworbenen neuromuskulären Störungen verwenden, können Sie diese hier aufführen.**

(Bitte nur ausführen, wenn Sie in Frage 3.1 die Antwortmöglichkeit "Andere, hier nicht genannte" ausgewählt haben)

3.3 **Gab es bei Ihnen im vergangenen Jahr eine klinikinterne Fortbildung zum Thema neuromuskuläre Störungen und assoziierte Komplikationen des Intensivpatienten?**

- ☐ Ja
- ☐ Nein

3. Diagnostik der ICU-AW [Fortsetzung]

3.4 **Gibt es zum jetzigen Zeitpunkt auf Ihrer Intensivstation ein strukturiertes diagnostisches Vorgehen zur Detektion einer ICU-AW (z.B. eine hausinterne SOP)?**

☐ Ja ☐ Nein

3.5 **Die aktuell gültige S3-Leitlinie Invasive Beatmung und Einsatz extrakorporaler Verfahren bei akuter respiratorischer Insuffizienz (AWMF Leitlinien-Register Nr. 001-021) empfiehlt die frühzeitige Sicherung der Diagnose ICU-AW durch die Anwendung eines systematischen Screenings auf der Intensivstation.**

Bitte geben Sie an, welche der nachfolgenden Methoden auf Ihrer Intensivstation für ein systematisches Screening zur Diagnosestellung der ICU-AW genutzt werden.

Bitte wählen Sie alle zutreffenden Antwortmöglichkeiten aus.

☐ Klinisch-neurologische Untersuchung
☐ Anwendung von selektiven Scores (z.B. Medical Research Council sum score, MRC-SS)
☐ Elektroneurographie (ENG) / Elektromyographie (EMG)
☐ Neuromuskulärer Ultraschall
☐ Labor-/ Biomarkerdiagnostik
☐ Muskel-/Nervenbiopsie
☐ Andere, hier nicht genannte
☐ Keine

3.6 **Wenn Sie andere Methoden für ein systematisches Screening zur Diagnosestellung der ICU-AW auf Ihrer Intensivstation verwenden, können Sie diese hier aufführen.**

(Bitte nur ausführen, wenn Sie in Frage 3.5 die Antwortmöglichkeit "Andere, hier nicht genannte" ausgewählt haben)

3.7 **Bitte geben Sie an, unter welchen Umständen Sie einen Patienten auf Ihrer Intensivstation aktuell auf das mögliche Vorliegen einer ICU-AW untersuchen.**

Bitte wählen Sie alle zutreffenden Antwortmöglichkeiten aus.

☐ Routinemäßig, im Rahmen der täglichen klinischen Untersuchung
☐ Wenn Grunderkrankung und Erkrankungsschwere dies wahrscheinlich machen
☐ Wenn der Patient über einen längeren Zeitraum eine reduzierte oder keine motorische Eigenaktivität zeigt
☐ Wenn der Patient einen erfolglosen Weaningversuch bei invasiver Beatmung hatte
☐ Andere, hier nicht genannte
☐ Keine

MUSTER

3. Diagnostik der ICU-AW [Fortsetzung]

3.8 **Wenn Sie Patienten auf Ihrer Intensivstation unter anderen Umständen auf das mögliche Vorliegen einer ICU-AW untersuchen, können Sie diese hier aufführen.**

(Bitte nur ausführen, wenn Sie in Frage 3.7 die Antwortmöglichkeit "Andere, hier nicht genannte" ausgewählt haben)

3.9 **In welchen zeitlichen Abständen führen Sie ein Screeningverfahren zur Diagnostik einer ICU-AW durch?**

- ☐ Einmal pro Aufenthalt des Patienten
- ☐ Einmal täglich
- ☐ Einmal pro Schicht
- ☐ Ein Screening wird nicht durchgeführt

3.10 **Wer sollte aus Ihrer Sicht ein Screeningverfahren zur Detektion der ICU-AW vorrangig durchführen?**

- ☐ Pflegepersonal
- ☐ Ärztliches Personal
- ☐ Physiotherapeuten

3.11 **Wer führt aktuell tatsächlich ein Screeningverfahren zur Detektion der ICU-AW auf Ihrer Intensivstation durch?**

- ☐ Pflegepersonal
- ☐ Ärztliches Personal
- ☐ Physiotherapeuten

MUSTER

4. Monitoring der ICU-AW

4.1 **Gibt es zum jetzigen Zeitpunkt auf Ihrer Intensivstation ein strukturiertes Konzept für die weiterführende Intensivbehandlung, wenn die Diagnose einer ICU-AW gestellt wurde (z.B. intensivierte Physiotherapie oder angepasste Weaningstrategien bei Beatmung)?**

☐ Ja
☐ Nein

4.2 **Führen Sie nach der Diagnosestellung einer ICU-AW weiterführende Diagnostik auf Ihrer Intensivstation durch?**

Bitte wählen Sie alle zutreffenden Antwortmöglichkeiten aus.

☐ Elektrophysiologische Untersuchungen (Elektroneuro-/ Elektromyographie)
☐ Neuromuskulärer Ultraschall
☐ Muskel-/ Nervenbiopsie
☐ Anmeldung eines neurologischen Konsils
☐ Labor-/ Biomarkerdiagnostik
☐ Andere, hier nicht genannte
☐ Keine

4.3 **Wenn Sie andere Untersuchungen zur weiterführenden Diagnostik nach Detektion einer ICU-AW auf Ihrer Intensivstation durchführen, können Sie diese hier aufführen.**

(Bitte nur ausführen, wenn Sie in Frage 4.2 die Antwortmöglichkeit "Andere, hier nicht genannte" ausgewählt haben)

4.4 **Welche der folgenden Methoden verwenden Sie zur Verlaufskontrolle der ICU-AW im Rahmen der Intensivbehandlung?**

Bitte wählen Sie alle zutreffenden Antwortmöglichkeiten aus.

☐ Klinisch-neurologische Untersuchung
☐ Anwendung selektiver Scores (z.B. Medical Research Council sum score, MRC-SS)
☐ Elektroneurographie (ENG) / Elektromyographie (EMG)
☐ Neuromuskulärer Ultraschall
☐ Labor-/ Biomarkerdiagnostik
☐ Muskel-/ Nervenbiopsie
☐ Andere, hier nicht genannte
☐ Keine

4. Monitoring der ICU-AW [Fortsetzung]

4.5 **Wenn Sie andere Methoden zum Monitoring des Krankheitsverlaufs einer ICU-AW auf Ihrer Intensivstation verwenden, können Sie diese hier aufführen.**

(Bitte nur ausführen, wenn Sie in Frage 4.4 die Antwortmöglichkeit "Andere, hier nicht genannte" ausgewählt haben)

4.6 **Welchen der nachfolgenden Scores verwenden Sie, um die körperliche Einschränkung Ihrer Intensivpatienten mit ICU-AW einzuschätzen?**

- ☐ Functional Independance Measure (FIM)
- ☐ Physical Function in the ICU Test (PFIT)
- ☐ Functional Status Score for ICU (FSS-ICU)
- ☐ Acute Care Index of Function (ACIF)
- ☐ Andere, hier nicht genannte
- ☐ Keine

4.7 **Wenn Sie andere Scores zur Erfassung der körperlichen Einschränkungen von Patienten mit ICU-AW auf Ihrer Intensivstation verwenden, können Sie diese hier aufführen.**

(Bitte nur ausführen, wenn Sie in Frage 4.6 die Antwortmöglichkeit "Andere, hier nicht genannte" ausgewählt haben)

5. Klinisch-therapeutische Konsequenzen

5.1 **Welche weiterführenden intensivmedizinischen Konzepte werden nach Diagnosestellung einer ICU-AW auf Ihrer Intensivstation umgesetzt?**

Bitte wählen Sie <u>alle zutreffenden</u> Antwortmöglichkeiten aus.

- ☐ Einleitung bzw. Intensivierung einer physiotherapeutischen Behandlung
- ☐ Anwendung neuromuskulärer elektrischer Stimulation
- ☐ Intensivierte Insulintherapie zur Vermeidung von Hyperglykämien
- ☐ Vermeidung von Muskelrelaxantien
- ☐ Vermeidung von Kortikosteroiden
- ☐ Reduktion der Sedativagabe
- ☐ Andere, hier nicht genannte
- ☐ Keine

5.2 **Wenn Sie andere intensivmedzinische Konzepte nach Diagnosestellung einer ICU-AW umsetzen, können Sie diese hier aufführen.**

(Bitte nur ausführen, wenn Sie in Frage 5.1 die Antwortmöglichkeit "Andere, hier nicht genannte" ausgewählt haben)

5.3 **Wie oft ist auf Ihrer Intensivstation eine physiotherapeutische Behandlung bei Patienten mit ICU-AW in der klinischen Routine realisierbar?**

- ☐ Mehrmals pro Schicht
- ☐ Einmal pro Schicht
- ☐ Einmal täglich
- ☐ In unregelmäßigen Abständen
- ☐ Gar nicht

5.4 **Wo sehen Sie aktuell Defizite in der Versorgung von Intensivpatienten mit ICU-AW?**

Bitte wählen Sie <u>alle zutreffenden</u> Antwortmöglichkeiten aus, die am ehesten zutreffen.

- ☐ In der Verfügbarkeit von diagnostischen / therapeutischen Möglichkeiten
- ☐ In der Zeit, die dem medizinischen Personal für die intensivierte Behandlung zur Verfügung steht
- ☐ In der Verfügbarkeit von Physiotherapeuten
- ☐ In der Verfügbarkeit von Pflegekräften
- ☐ Im spezifischen Fachwissen des medizinischen Personals
- ☐ Andere, hier nicht genannte
- ☐ Keine Defizite vorhanden

MUSTER

5. Klinisch-therapeutische Konsequenzen [Fortsetzung]

5.5 **Wenn Sie andere Defizite in der Versorgung von Intensivpatienten mit ICU-AW sehen, können Sie diese hier aufführen.**

(Bitte nur ausführen, wenn Sie in Frage 5.4 die Antwortmöglichkeit "Andere, hier nicht genannte" ausgewählt haben)

5.6 **Werden Angehörige über mögliche längerfristig bestehende körperliche Einschränkungen des Patienten mit ICU-AW aufgeklärt?**

- ☐ Ja
- ☐ Nein

5.7 **Wird die Diagnose einer neu erworbenen neuromuskulären Störung im Rahmen der Intensivtherapie bzw. ICU-AW in der Epikrise des Patienten aufgeführt?**

- ☐ Ja
- ☐ Nein
- ☐ Nicht regelhaft

5.8 **Sollte aus Ihrer Sicht eine standardisierte Empfehlung im Sinne einer spezifischen Leitlinien zu Diagnostik, Monitoring und klinisch-therapeutischen Ansätzen bei ICU-AW entwickelt werden?**

- ☐ Ja
- ☐ Nein

5.9 **Abschließend möchten wir die Umfrage mit einer Testfrage zum Thema Diagnostik der ICU-AW beenden.**

Welche der nachfolgenden Aussagen trifft NICHT auf den Medical Research Council sum score (MRC-SS) zu:

- ☐ Die Fähigkeit zur Mitarbeit des Patienten (bewertet mittels initialem Score of five questions) spielt bei der Durchführung des MRC-SS keine Rolle
- ☐ Standardisierte Untersuchungsbedingungen sollten stets vorhanden sein
- ☐ Der MRC-SS kann auch Vorliegen einer Hemiparese oder Monoparese einer Extremität angewendet werden
- ☐ Auch beatmete Patienten können mittels MRC-SS untersucht werden

MUSTER

5. Klinisch-therapeutische Konsequenzen [Fortsetzung]

5.10 **Haben Sie noch abschließende Anmerkungen zur Umfrage?**
